# An Interesting Case of Klebsiella Pneumoniae Pylephlebitis

**DOI:** 10.7759/cureus.21297

**Published:** 2022-01-16

**Authors:** Sneha Adidam, Lorenzo Leys, Siham Mahgoub

**Affiliations:** 1 Internal Medicine, Howard University Hospital, Washington DC, USA; 2 Pulmonology and Critical Care, Mount Sinai Medical Center, New York, USA; 3 Infectious Disease, Howard University Hospital, Washington DC, USA

**Keywords:** klebsiella pneumoniae, septic portal vein thrombosis, portal vein thrombosis, abdominal pain, portal vein pylephlebitis, pylephlebitis

## Abstract

Pylephlebitis is defined as suppurative thrombophlebitis of the portal venous system. It is a rare condition that can be fatal if left untreated. It is usually caused by polymicrobial bacteria, most commonly *Escherichia coli* and *Streptococcus* genus. *Klebsiella pneumoniae* have been identified but the literature does not suggest a percentage of cases caused by this organism. The presentation includes abdominal pain, signs of sepsis and even septic shock. We present a case of a middle-aged female with *K. pneumoniae* bacteremia and pylephlebitis, with portal vein thrombosis visualized on an abdominal ultrasound. Although the patient was treated with broad-spectrum antibiotics and anticoagulation, she succumbed to multiorgan failure and septic shock on day two of intensive care.

## Introduction

Pylephlebitis is a condition of thrombophlebitis of the portal venous system which can be secondary to an intraperitoneal focus of infection [1]. It may present with abdominal pain, fever, chills, leukocytosis and can be diagnosed with abdominal ultrasonography or other imaging such as CT or MRI of the abdomen. *Klebsiella pneumoniae* is one of the organisms that cause pylephlebitis and may cause metastatic infections involving the lungs, urinary tract, and liver [2]. Some of the invasive infections caused by *K. pneumoniae *such as bacteremia, as seen in the index case, has been associated with hypermucoviscous phenotype that was first identified in 2004 [3]. Herein, we present a rare case of the fatal disorder of pylephlebitis in a patient found to have *K. pneumoniae* bacteremia with portal vein thrombosis. 

## Case presentation

A sixty-year-old female with past medical history of hypertension, myocardial infarction, and right femur fracture with hardware placement approximately two years prior, was brought into the emergency department because of abdominal pain, chest discomfort and right lower extremity pain which began on the day of presentation. Initial vitals were temperature 97.3 degrees Fahrenheit, heart rate 103 beats per minute, blood pressure 137/61, respiratory rate 21 with a saturation of 100% on room air. On presentation, she was ill-appearing and lethargic. Physical exam revealed tachycardia. On palpation, she had right and left lower quadrant and suprapubic abdominal tenderness without guarding or rebound tenderness and diffusely diminished breath sounds. There was no costovertebral angle tenderness. There was an edematous, indurated, tender right thigh with ecchymosis. Laboratory studies yielded a lactic acid of 9.3 mm/L that increased to 11.4 mm/L even after starting fluid resuscitation and significant D dimer elevation to 9.69. Other significant laboratory values are listed in Table 1.

**Table 1:** Table showing significant laboratory values ALT: Alanine transaminase, AST: Aspartate aminotransferase, ALP: Alkaline phosphatase, PT: Prothrombin time, PTT: Partial thromboplastin time

Lab parameter	Admission	Day 2	Normal Range
Lactic acid	9.3	10.4	0.5-2.2 mm/L
White cell count	3.35	6.83	3.2-10.5 x 10^ 9
Platelet	47	15	177-406 x 10^ 9
Haemoglobin	11.3	6.9	12.1-15.9 g/dL
ALT	20	77	0-55 IU/L
AST	52	479	0-50 IU/L
ALP	32	30	30-130 IU/L
D-dimer	7.14	9.69	0-0.48 ug/ml
PT	28	41.6	12.5-14.5 sec
PTT	50.5	90.9	25-35 sec
Fibrinogen	173	103	237-507 mg/dL
Fibrinogen degradation product	10-40	>40	1.25-10 uG/mL
Total bilirubin	2.7	4.2	0.2-1.2 mg/d L

**Figure 1:**
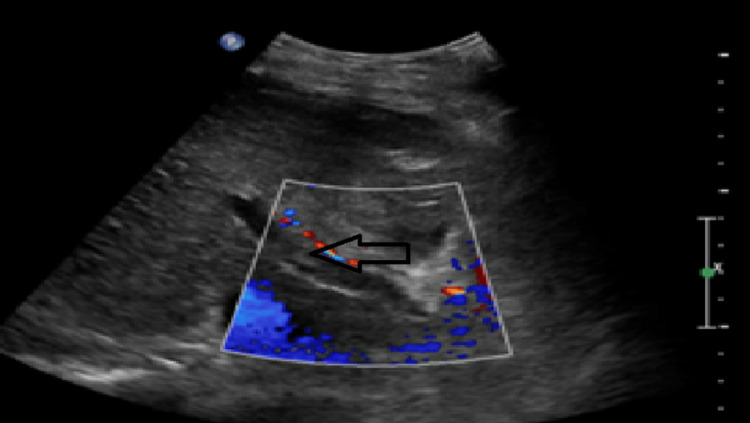
Abdominal ultrasound showing no flow detected in the portal vein suggestive of portal vein thrombosis.

Urinalysis showed trace leukocytes, moderate ketones, 0-1 white blood cells and significant glucosuria. Although the urinalysis was abnormal, the patient’s clinical status deteriorated rapidly and so, urine culture was not done. Urine toxicology showed cocaine and opioid use. Blood cultures grew pan-sensitive *K. pneumoniae*. Chest X-ray showed hypoinflation of the lungs, interstitial oedema and cardiomegaly. She was initially assessed to have a possible cardiac event and was admitted to the cardiology unit. However, despite fluid resuscitation, her vitals deteriorated within three hours with a heart rate of 101 beats per minute, blood pressure 86/49 and respiratory rate of 24 breaths per minute with a saturation of 84% on 4 litres/minute of oxygen via nasal cannula. She was transferred to the ICU for septic shock. The patient was stabilized with a nonrebreather mask, fluid bolus and vasopressors. Broad-spectrum antibiotics including vancomycin and piperacillin-tazobactam were initiated. Considering hypoxia, tachycardia, right leg oedema and erythema, therapeutic intravenous anticoagulation was initially started for concern of deep vein thrombosis and pulmonary embolism. Transthoracic echocardiogram did not reveal any valvular lesions. The lower extremity Doppler ultrasound ruled out deep vein thrombosis but an abdominal ultrasound revealed no flow in the portal vein suggesting portal vein thrombosis (as shown in Figure 1 below). Gall stones were seen in the gallbladder with negative Murphy’s sign. Furthermore, the patient was unstable for a CT scan of the abdomen and pelvis on this admission. The patient remained hypotensive requiring three pressors. She then developed worsening sepsis, hypoglycemia, acute encephalopathy, acute respiratory distress syndrome and disseminated intravascular coagulation. The patient continued to deteriorate and expired on day two of her ICU admission.

## Discussion

In this case, we discuss the systemic manifestations of *K. pneumoniae.* *Klebsiella pneumoniae-*causing pylephlebitis has a mortality of 11% to 32% [4]. The data for pylephlebitis was difficult to find. However, according to one article by Condat et al., the incidence of patients with identified pylephlebitis increased to 56% after the year 1994 compared to 7% before 1990 [5]. Invasive cases secondary to *K. pneumoniae* infection were first limited to Southeast Asia and South Africa. Now, it is evident that this invasive virulent strain has crossed geographical borders [6]. Some risk factors for *K. pneumoniae* bacteremia include malignancy, dialysis-dependence and even non-modifiable risk factors such as age and gender, with the elderly and males at higher risk [7]. Our patient was a middle-aged female with none of the risk factors mentioned above. She also had no recent travel history to Asia or Africa. Furthermore, the most common focus for such infection was appendicitis before the widespread use of antibiotics and now this has transitioned to diverticulitis [8]. Despite a robust attempt to delineate the source of the bacteremia and pylephlebitis, it was challenging. Presumably, the source in the index case was a soft tissue infection or a hepatobiliary source. Furthermore, studies have identified nearly one third of patients with hepatic or portal vein thrombophlebitis, also having *Klebsiella* liver abscess [9]. However, ultrasound studies in our index patient did not show any development of an abscess. Although our patient’s presentation defied the odds of developing *K. pneumoniae* pylephlebitis based on demographics and risk factors, she was found to have positive blood cultures growing pan-sensitive *K. pneumoniae*. Kanellepoulou et al. described that positivity of blood cultures or other sites was approximately 77% in pylephlebitis. Also, 50% of cases were polymicrobial, and other organisms described were *Escherichia coli*, *Bacteroides fragilis* and *Streptococcus* species [10]. However, another article demonstrated that in a systematic review, 75% of cases were acquired from the community and monomicrobial [11]. This review also revealed that fever, elevated white blood cell count and abdominal pain were features found in all patients with pylephlebitis [11]. 

Our patient was treated with broad-spectrum antibiotics including vancomycin and piperacillin/tazobactam and unfractionated heparin drip for anticoagulation. Surgical intervention was not considered and was precluded as the patient was critically ill and coded by day two of intensive care unit admission. The treatment of choice of pylephlebitis include metronidazole and third-generation cephalosporin or a carbapenem to cover anaerobic, aerobic, and gram-negative bacilli. The duration of therapy can be at least four weeks [12]. There is no consistent data on the use of anticoagulation. It is noted that there are risks and benefits to therapeutic anticoagulation, as in any other case of thrombosis. Resolution of portal vein thrombosis has been seen in 58% of patients after use of anticoagulation as opposed to only 21% in those who did not use anticoagulation [13]. Recurrence has been seen in 6% to 40% of *Klebsiella,* in which the species was identified as *Klebsiella oxytoc*a [14]. However, our patient was noted to have a possible soft tissue source of infection which deemed her a poor candidate for surgical drainage given the rapid decline and death in this case, leaving very minimal time for further intervention.

## Conclusions

This case serves to explore *K. pneumoniae* bacteremia, complicated by pylephlebitis, which may be found not only in specific parts of the eastern hemisphere (Asia/Africa) but also in the United States. Clinicians should maintain a high index of suspicion for possible systemic or portal circulation thrombosis. We propose to continue the use of antibiotics, anticoagulation, and even surgical management in appropriate cases. It is our humble attempt to continue to study and propagate the early recognition of the many manifestations of invasive *K. pneumoniae*.
